# Impact of Tobacco Smoking and Type-2 Diabetes Mellitus on Public Health: A Cerebrovascular Perspective

**DOI:** 10.4172/2329-6887.S2-e003

**Published:** 2015-11-12

**Authors:** Shikha Prasad, Luca Cucullo

**Affiliations:** 1 Department of Pharmaceutical Sciences, 1300 S Coulter St, Amarillo TX 79106-1712, USA; 2 Center for Blood-Brain Barrier Research, Texas Tech University Health Sciences Center, Amarillo, TX 79106, USA

## Editorial

Tobacco smoke (TS) is accountable for ≈ 434,000 casualties/year in the US and is the leading cause of preventable death. Even though there has been a marginal decline in smoking during recent years, the fact that ≈ 18% of the US adult population are current smokers is alarming [1]. In 2007 diabetes was the 7th leading cause of death in the US and increasing at an alarming rate. One in every three U.S. adults is projected to suffer from diabetes by 2050 [2]. Smoking is a major risk factor for diabetes [3], with 12% of Type-2 Diabetes Mellitus (T2DM) cases being attributed to tobacco smoke (45% higher in men, 74% higher in women in comparison to non-smokers) [4-6]. Both active and passive smoking not only causes glucose intolerance [7], but also significantly increases the risk of diabetes. Major pathological changes in diabetic patients such as insulin resistance and high levels of glycated hemoglobin (HbA1c) have also been reported in smokers [5]. Similarly to TS, the risk of myocardial infarction and stroke is 4-fold higher in 2DM independently of other known risk factors [8]. Both T2DM and TS have independently been reported to enhance the risk of cerebrovascular and neurological disorders, however the pathophysiological mechanisms underlying these cerebrovascular disorders remain elusive. CS contains over 4000 chemicals including nicotine and various reactive oxygen species (ROS) (e.g., H_2_O_2_, epoxides, nitrogen dioxide, peroxynitrite -ONOO-, etc. [9,10] which pass through the lung alveolar wall and raise systemic oxidative stress OS [11]. At the cerebrovascular level this promotes oxidative damage and BBB breakdown via tight junction (TJ) modification and activation of pro inflammatory pathways [12,13]. Under normal conditions, ROS are scavenged by antioxidant vitamins such as ascorbic acid and α-tocopherol [14-17] or intracellularly converted into less reactive molecules by superoxide dismutase (SOD), catalase, and glutathione peroxidase (GSH-Px) [18]. Both acute and chronic nicotine exposure has even shown to reduce stroke induced enhancement in GLUT1 transport function and expression at the BBB in a focal brain ischemia model [19]. However, chronic exposure to active and passive smoking can overwhelm these protective mechanisms. Elevated levels of WBC, primarily neutrophils and monocytes, are observed in smokers [20]. In particular, neutrophils, which secrete free radicals, elastase and collagenase [21], are thought to contribute directly to endothelial cells (EC) injury. Platelet activation is also frequently observed in smokers [22] and confirmed in vitro and in vivo studies [23].

Chronic hyperglycemia, a pathogenic alteration characteristic of T2DM, also causes endogenous ROS increase by inhibiting glycolysis and promoting the formation of harmful intermediates (such as advanced glycation end products (AGEs) and protein kinase-C pathway (PKC) isoforms) which have DNA and protein damaging effects [24-26]. T2DM causes endothelial dysfunction leading to BBB impairment and loss of barrier integrity [26].

## Effects of Oxidative Stress by Hyperglycaemia

Glucose is the primary source of energy for the brain, which consumes around 25% of the total glucose available in the body. Diabetes is generally characterized by hyperglycemia followed by a sharp decline in plasma glucose levels upon administration of insulin injection/anti-diabetic medication [26]. A state of hyperglycemia particularly damages endothelial cells and those similar where the glucose transporter expression does not decline in proportion to the excess glucose available, thereby leading to an increase in intracellular glucose [24]. Excess glucose and free fatty acid flux from adipocytes to macrovascular endothelial cells resulting in mitochondrial overproduction of ROS. Increased ROS levels activate poly-ADP-ribose polymerase-1(PARP-1) causing an inhibition of glyderaldehyde-3-phosphate dehydrogenase (GAPDH) by poly-ADP-ribosylation, thereby impeding the progress of glycolysis and increasing the presence of glycolytic intermediates. These intermediates enter into several by-pathways like polyol, hexosamine, protein kinase-C (PKC) and advanced glycation end products (AGE) pathways. The resulting effects translate into either utilization of important enzymes like aldose reductase or formation of unwanted intermediates like AGEs and PKC isoforms, which have damaging effects on DNA such as DNA strand breakage [27-30], and nitric oxide (NO) and antioxidant depletion which similarly to tobacco smoke can impact the viability of the cerebrovascular system and promote inflammation. Recent observations suggest that ROS are key mediators of BBB breakdown [31].

## Role of HMGB1 in Oxidative Stress-Dependent BBB Damage

HMGB1 is a prototypic damage-associated molecular pattern (DAMP) protein highly secreted by activated macrophages and monocytes as a cytokine mediator of inflammation. This DNA-binding nuclear protein is released both passively during cell death and actively following cytokine stimulation. It is also implicated in both infectious and sterile inflammatory disorders [32-36] affecting the central nervous system (CNS) such as in Parkinson’s disease (PD) [37], multiple sclerosis (MS) [38,39], ischemic stroke [40], traumatic brain injury (TBI) [41] and Alzheimer’s disease - AD [42-44]. HMGB1 activates cells by differential engagement of several membrane receptors including advanced glycation end products (RAGE), toll-like receptor 2 (TLR2), and TLR4 which are primarily responsible for HMGB1 pro-inflammatory activity and BBB impairment [45,46]. Specific to the proposed work, several studies have clearly outlined the role of OS in the development of microvascular and cardiovascular complications of 2DM [47]. These underpin the primary role of HMGB1 [48,49] and the receptor for advanced glycation end-products (RAGE) [50,51] in the onset of 2DM-mediated inflammatory vascular damage [52,53] and BBB dysfunction [13,26]. Along a similar line, TS-dependent cerebrovascular damage has been linked to smoking-dependent generation of ROS and oxidative stress [12,54-56] which may support the possible activation of a similar pathogenic course also involving HMGB1 [57] and RAGE activation leading to the development of CNS degenerative disorders [43]. HMGB1 can directly impact BBB integrity thereby exposing the brain to inflammatory, toxic or other circulating substances. By directly penetrating the brain microvasculature, HMGB1 can then bind to glial and/or neuronal receptors on microglia and astrocytes, leading to changes in their functional phenotype [58].

## Neuroprotective Role of Nrf2 in oxidative stress-dependent BBB damage

Based on a recent gene array study by our lab [59], alteration of Nrf2-ARE pathways were among the most predominant gene transcription/translation changes we observed in human BBB microvascular endothelial cells exposed either to TS or 2DM-like altered glycaemic conditions (hyperglycaemia) [60]. These include Nrf2 nuclear translocation in BBB endothelium; upregulation of Nrf2-ARE dependent Phase I and II detoxification genes; and upregulated synthesis and activity of various antioxidant enzymes. Further, emerging evidence indicates a neuroprotective role of nuclear-factor (erythroid derived 2) related factor-2 (Nrf2) signaling in preventing cerebrovascular dysfunction associated with several CNS pathologies. In fact, Nrf2 activation alleviates early brain injury and cognitive dysfunction in experimental models of subarachnoid hemorrhage and traumatic brain injury [61]. Defective Nrf2-dependent redox signaling has been implicated in microvascular dysfunction in both 2DM [62] and CS [63]. From this perspective drugs enhancing Nrf2-ARE which promote increased translocation of Nrf2 in the nucleus, increased degradation of its binding Keap1, increase in downstream activity such as increased GSH and/or enzymes such as NQO-1, HO-1 [64-66] may hold promise in future to reduce the BBB injury outcome in chronic smoking and T2DM population and perhaps provide therapeutic benefits for the treatment of neurovascular disorders (e.g., stroke) where oxidative stress and inflammation play a prodromal role.
